# Synthesis and Evaluation of Chitosan-Vitamin C complex

**DOI:** 10.4103/0250-474X.57284

**Published:** 2009

**Authors:** X. L. Tian, D. F. Tian, Z. Y. Wang, F. K. Mo

**Affiliations:** School of Pharmacy, Shenyang Pharmaceutical University, 110016, Shenyang, China

**Keywords:** Polymer, chitosan, vitamin C, intrinsic viscosity, antioxidation

## Abstract

Chitosan is a biocompatible, biodegradable and non-toxic polysaccharide polymer. It dissolves in water only if the pH is lower than 6.5. To extend its range of application, many water-soluble derivatives have therefore been prepared. In this research, chitosan-vitamin C complex was synthesized and characterized with Fourier transformed infrared spectroscopy, differential scanning calorimetry and ^1^H-NMR. The solubility of chitosan-vitamin C complex in distilled water was greatly improved. The •O_2_^−^ scavenging activity of chitosan-vitamin C complex was compared with chitosan and vitamin C by measuring the auto-oxidation rate of pyrogallic acid. Results showed that the scavenging activity on •O_2_^−^ by chitosan-vitamin C complex was stronger than that by CS. At low concentrations (< 0.05 mg/ml), the scavenging activity of chitosan-vitamin C complex was stronger than that of vitamin C, but after certain concentrations (>0.1mg/ml), its scavenging activity was lower than that of vitamin C.

Chitosan (CS), a partially deacetylated form of chitin, is an amino-containing basic polysaccharide formed primarily of repeating units of β-(1,4)-2-amino-2-deoxy-D-glucose. With a pKa of 5.5-6.5, CS is soluble and forms the cationic polymer in aqueous acidic medium below pH 6.5 in the presence of a small amount of acids such as inorganic acid (HCl and HNO_3_) and organic acid (AcOH and lactic acid) due to the fact that the amino groups dispersed on the structure are protonated. It is insoluble in water and precipitates above pH 6.5 by the addition of alkali solution[1]. Because of its favorable physicochemical and biological properties such as being gel-formable at low pH ranges, antibacterial, biodegradability, biocompatible, epithelial permeation enhancement, mucoadhesive and non-toxic, CS is considered as an ideal excipient that can potentially be used as a polymeric carrier in targeted drug delivery and sustained release system[2]. The application of CS in pharmaceutical respect includes gel formulations, gastric float formulations, bioadhesive formulations and colon-targeted formulations[3,4].

The application of CS in biomedical fields is limited owing to the poor solubility in physiological media. Through chemical modification, CS derivatives of specific functions (i.e. improved solubility and bioactivity) can be obtained by introducing active groups on to the hydroxyl (C3 and C6) and amino groups (C2) of CS molecules. Studies suggest that amino groups on the surface of CS chains are reactive enough to react with a number of acid chlorides, acid anhydrides and aldehydes[5].

In biological systems, vitamin C (VC) plays the role of an effective antioxidant due to the presence of the enediol moiety. It also serves as a cofactor in hydroxylation reactions and scavenges reactive oxygen species. The biochemical functions of VC, especially its functions in antivirus and antitumor are of increasing interests[6]. However the use of VC is limited by its physical and chemical instability. VC is a six-carbon keto-lactone, which contains four hydroxyls and a lactone. It is highly unstable and very easy to get oxidized and changes to dehydroascorbic acid when exposed to light, air and elevated temperature. To increase the stability of VC, various derivatives have been synthesized, including the metal salts (Na, Ca salts), ethers, esters and the polysaccharide derivatives[7].

CS contains amino groups which are protonatable in the acidic media, whereas VC contains acidic hydroxyl functionality, thus allowing the formation of a complex through ionic interaction. The synthesis of salts of VC with different substituted amines is also supported by literature[8]. In this research the synthesis of CS-VC complex (CSVC) was investigated, which was further characterized by FT-IR, DSC and ^1^H-NMR. The solubility and •O_2_^−^ scavenging activity between CS and CSVC were compared.

## MATERIALS AND METHODS

CS was purchased from Zhejiang Golden Shell Biochemical Co., Ltd. and used without further purification. The viscosity molecular weight was 8.56×10^5^ Da and the degree of deacetylation was 84% as measured on an acid-base titration method. VC was from Northeast Pharmaceutical Group Co., Ltd. The double distilled water was used for all experiments. Pyrogallic acid was from Tian Jin Bodi Chemicals Company Ltd. Isopropyl alcohol was of the analytical grade.

### Synthesis of CS-vitamin C complex (CSVC):

A 250 ml, three-necked round bottomed flask was charged with 6.0 g of CS and 60 ml of isopropyl alcohol, and the flask was fitted with a subsurface nitrogen feed to remove dissolved oxygen from solutions. While stirring the slurry under nitrogen for 1 hour, a solution of 6.62 g VC in 25 ml of distilled water was prepared. This solution was then added to the CS slurry by syringe, and after 30 min, a solution of 26 ml of isopropyl alcohol and 11 ml of distilled water was added. After stirring for 2 additional h, the polymer was recovered under nitrogen. The polymer was washed under nitrogen once with a solution of 160 ml of isopropyl alcohol and 40 ml of water, and once with 200 ml of isopropyl alcohol. The light tan solid was brought to near dryness under a nitrogen stream. Finally, the product was dried at 40^°^ under vacuum for 24 h.

### Analysis by Fourier transformed infrared spectroscopy (FT-IR) and differential scanning calorimetry (DSC):

The infrared absorption spectra were obtained using a Bruker FT-IR spectrophotometer over the wave number range between 4000 and 400 cm^−1^. All powder samples were compressed into KBr disks for the FT-IR measurements. Thermal analysis was carried out using DSC60 Differential Scanning Calorimeter (Shimadzu Co., Japan). Approximately 2 mg of samples were hermetically sealed in an aluminum pan and heated from 30^°^ to 300^°^ at a scanning rate of 10 ^°^/min.

### Analysis by ^1^H-NMR:

The ^1^H-NMR spectra of CS and CSVC were obtained from 1% solution in 1% CF_3_COOH/D_2_O and D_2_O, respectively using a Bruker ARX-300 MHz spectrometer. The ^1^H-NMR spectrum of VC was obtained using D_2_O as the solvent. Chemical shifts were reported in ppm.

### Apparent solubility testing:

According to the general notices of Chinese Pharmacopoeia (2005 Edition), 0.1 g of CSVC was added into 1 ml and 10 ml of different solvents respectively and stirred vigorously at 25±1^°^ for 24 h to observe its apparent solubility based on the transparency of solutions.

### Antioxidation activity testing[9–11]:

The •O_2_^−^ scavenging capability of different samples was assayed by measuring the inhibition of auto-oxidation rate of pyrogallic acid. The blank solution was prepared as follows: Tris-HCl buffer (0.05 M, 4.5 ml, pH 7.2) was added into a 10 ml test tube followed by 1.1 ml of deionized water and vortexed. The UV/Vis absorbance of solutions containing pyrogallic acid without and with the test samples added were determined according to the following procedure: Tris-HCl buffer (0.05 M, 4.5 ml, pH 7.2) was added into a 10 ml test tube. 1 ml deionized water or 1 ml test sample solution at different concentrations was added under vortex. Then pyrogallic acid (10 mM, 0.1 ml) was added. The mixture was vortexed evenly and the absorbance at 325 nm was measured immediately against the blank solution at 60 seconds intervals for 4 minutes. The auto-oxidation rate constant of pyrogallic acid before and after the addition of test sample (k_0_ and k_1_ respectively) was calculated from the slope of curve of absorbance verses time. The antioxidation rate of test samples was calculated according to the following Eqn, antioxidation rate =(k_0_−k_1_/k_0_)×100%

### Statistics:

Results were depicted as mean±SD from at least three measurements. Significance between the mean values was calculated using ANOVA one-way analysis. Probability values *P*<0.05 were considered significant.

## RESULTS AND DISCUSSION

**Fig. 1:**
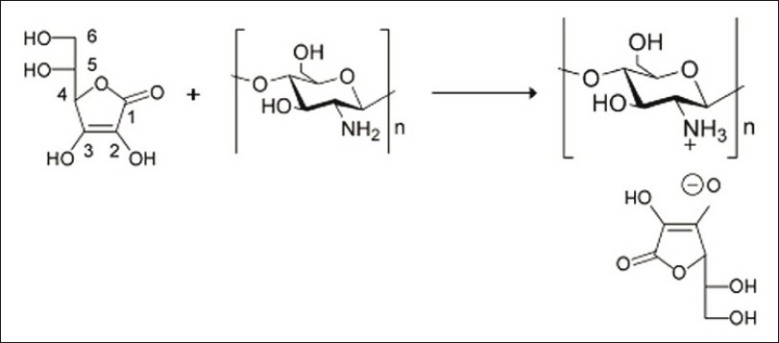
Possible reaction mechanism between CS and vitamin C

The product formed is CS-vitamin C complex, which is different from CS-vitamin C mixture in that no protonation of CS occurs in the latter[12]. VC presents several electrophilic groups. It contains four hydroxyl groups in positions 2, 3, 5 and 6 with different acidities allowing acid–base reactions. The -OH in position 3 is the more acidic one (pKa = 4.2), while the hydroxyl in position 2 has a pKa of 11.6, and those in positions 5 and 6 behave as secondary and primary alcohol (pKa ≈17 and 16, respectively)[13]. The acidic hydroxyl in position 3 of VC was expected to react with the amino group of CS, converting it into ammonium ions. The possible reaction mechanism was shown in fig. 1.

VC also presents a lactone structure, which can be subject to ring opening in the presence of amine. Comparison of the UV/Vis absorption spectrum of VC with that of CSVC indicated quite similar behavior at λ_max_ = 242 nm, clearly demonstrating the presence of VC moiety in the polymeric chains of CSVC.

**Fig. 2:**
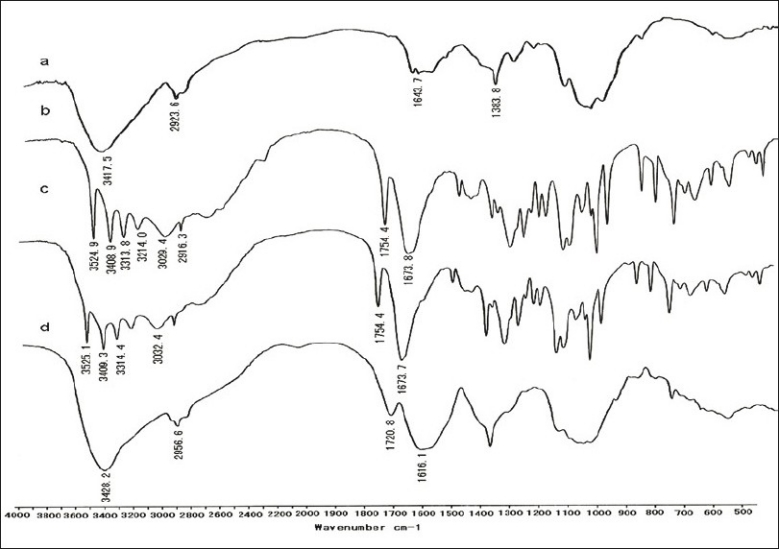
FT-IR spectra of CS, vitamin C, CS-vitamin C mixture and complexes. FT-IR spectra of a. chitosan (CS), b. vitamin C, c. CS-vitamin C mixture and d. CS-vitamin C complexes.

The FT-IR spectra of CS, VC, CSVC mixture and CSVC complex were shown in fig. 2. In fig. 2a, a characteristic strong peak at 3417.5 cm^−1^ was attributed to overlapping stretch vibrations of –NH_2_ and –OH groups and the peak for amide I (C=O) at 1643.7 cm^−1^ was seen. The aliphatic C-H stretching vibration of the polymer backbone occurred at 2923.6 cm^−1^[14]. The weak deformation vibration of C-CH_3_ appeared at 1383.8 cm^−1^, indicating a higher degree of deacetylation of CS used in this experiment[15]. The four peaks in fig. 2b from 3524 cm^−1^ to 3214 cm^−1^ were attributed to the four –OH groups at C_6_, C_3_, C_5_, C_2_, respectively. The stretch vibration of lactone C=O forming intramolecular H-bond occurred at 1754.4 cm^−1^ and that of lactone C=O forming intermolecular H-bond occurred at 1673.8 cm^−1^[16]. Figs. 2c and 2d showed quite different FT-IR spectra from each other, demonstrating the formation of complex between CS and VC. The peak at 1754.4 cm^−1^, which was the stretch vibration of lactone C=O forming intramolecular H-bond in VC, was shifted to 1720.8 cm^−1^ at a reduced intensity. It could be seen that new absorption band characteristic of bending vibration of -NH_3_^+^ appeared at 1616.1 cm^−1^. This result suggested that the -NH_2_ groups on the CS chains were protonated by the H^+^ supplied by VC[17]. The decrease of peak at 3428.2 cm^−1^ in fig. 2d indicated the reduction of free –NH_2_ groups after the formation of CSVC[18].

**Fig. 3:**
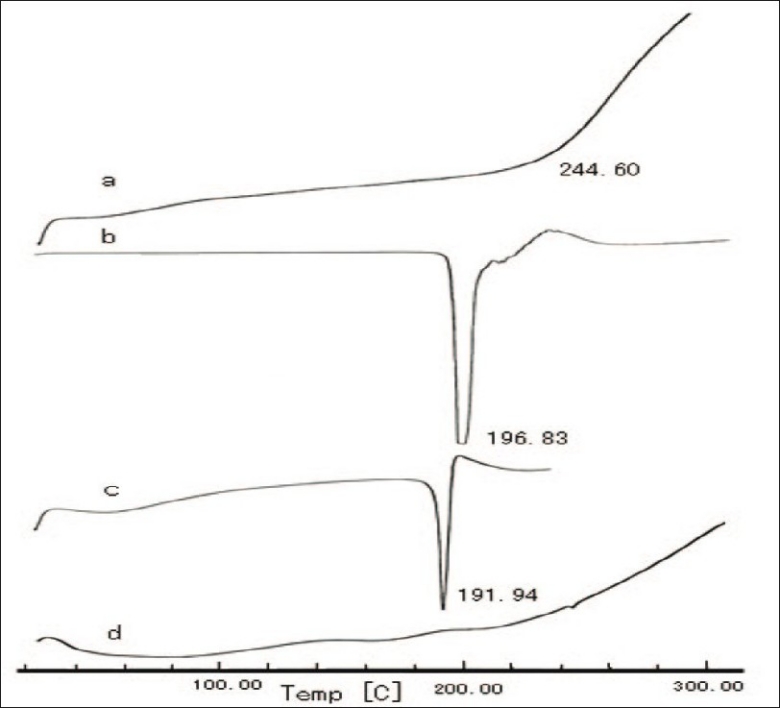
DSC thermographs of CS, vitamin C, CS-vitamin C mixture and complexes. The differential scanning calorimeteric thermographs of a. chitosan (CS), b. vitamin C, c. CS-vitamin C mixture and d. CS-vitamin C complexes

The DSC measurements were performed to investigate changes in the physical state of materials. As shown in fig 3a, the temperature at about 244.6^°^ was the onset temperature of decomposition for CS[19]. In figs. 3b and 3c, VC showed the melting point at 196.83^°^, which was decreased to 191.94^°^ in the CSVC mixture (2:1, g:g). However in CSVC fig. 3d, the endothermic peak of VC could not be seen. Therefore DSC testing results indicated the formation of complex between CS and vitamin C through the ionic interaction.

**Fig. 4:**
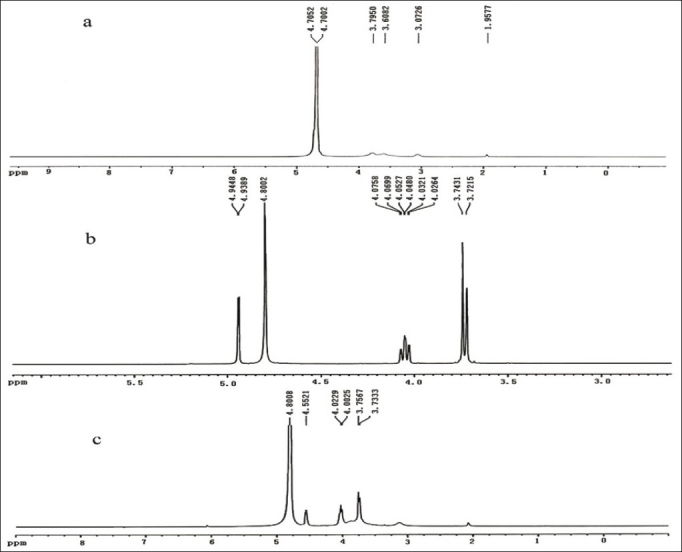
^1^H-NMR spectra of CS, vitamin C and CS-vitamin C complexes ^1^H-NMR spectra of chitosan (CS, a), vitamin C (b) and CS-vitamin C complexes (c)

The ^1^H-NMR spectrum of CS (fig. 4a) exhibited typical peaks, including the methyl protons from the survival N-acetylglucosamine unit at 1.9577 ppm (-COCH_3_) and the proton on the carbon bearing amino groups at 3.0726 ppm (H-2). The signals at 3.6082-3.7950 ppm were corresponding to the ring methenyl protons of CS backbones (H-3, H-4, H-5, H-6)[20,21]. The chemical shifts in the ^1^H-NMR spectrum of VC (fig. 4b) were assigned as follows: 4.94 ppm (CH-ring), 4.02-4.07 ppm (-CH) and 3.72-3.74 ppm (-CH_2_)[22,23]. In fig. 4c, the ^1^H-NMR spectrum of CSVC showed the characteristic peaks of CS and VC with certain chemical shifts because of the ionic interaction. The signal at 4.55 ppm was characteristic of the hydrogen at the C4 position of the VC ring, which appeared at 4.94 ppm before the formation of CSVC.

**Fig. 5:**
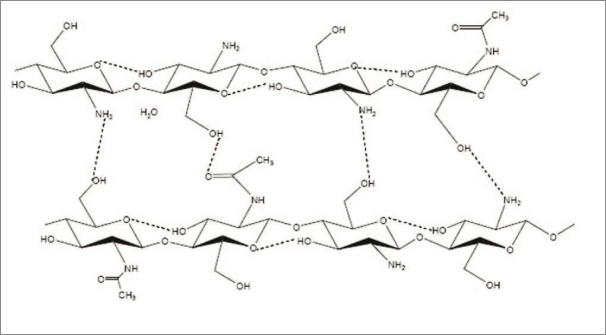
Crystalline structure of CS

Although the polymer backbone consists of hydrophilic functional groups, CS is normally insoluble in water and most common organic solvents (e.g. DMSO, DMF, NMP, alcohols and pyridine). The insolubility is a result of its extensive intramolecular and intermolecular H-bonding between the chains and sheets of CS as shown in fig. 5[24]. Our tests showed that CSVC was also insoluble in common organic solvents, such as DMSO, DMF, methanol, ethanol, acetonitrile and chloroform. However the solubility of CSVC in distilled water was greatly improved because most of the intermolecular H-bonding formed between –OH and –NH_2_ groups among CS molecules was broken down. Solubility tests showed that 0.1 g of the complex formed viscous and transparent gel in 1 ml of distilled water. A viscous solution was formed when 0.1 g of the complex was added into 10 ml of distilled water.

Pyrogallic acid can autooxidize in the alkaline condition to produce colored intermediate and •O_2_^−^. The colored intermediate showed maximum UV-absorption at 325 nm with a relatively constant production velocity within 4 min. The rate constant of this auto-oxidation reaction was dependent on •O_2_^−^ concentration because •O_2_^−^ could catalyze this auto-oxidation reaction. If samples with scavenging capacity on •O_2_^−^ were added into the system, the production of colored intermediate would be decreased, showing lowered UV-absorption. The lower the absorbance, the better the scavenging effect on •O_2_^−^.

**Fig. 6:**
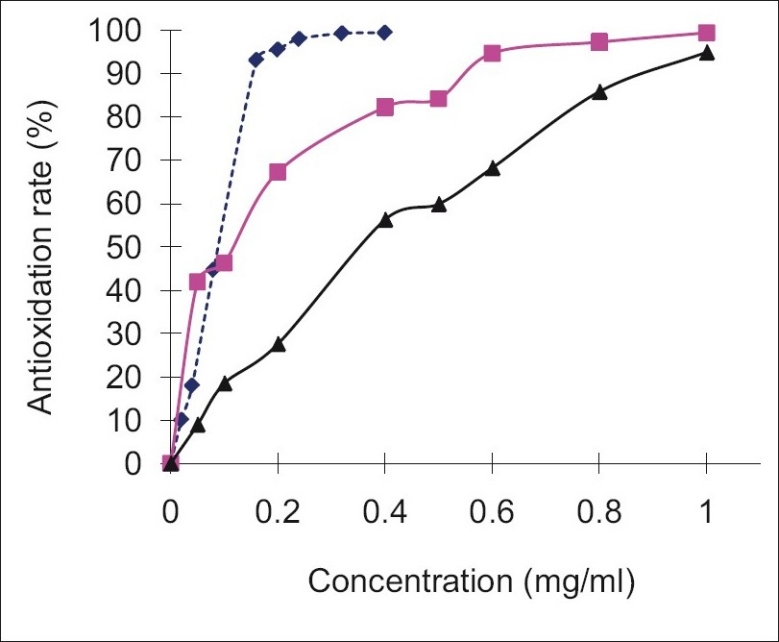
Antioxidation rates of CS, CSVC and VC. Antioxidation rates of chitosan (CS, –▲–), chitosan vitamin C complex (CSVC, –▪–) and Vitamin C (VC,…◆…)

Recently, the antioxidation activity of CS and its derivatives has attracted much attention. The active hydroxyl and amino groups in the polymer chains are the origin of the scavenging ability of CS[25]. Fig. 6 showed that the inhibitory effect of samples were concentration related, which increased as the sample concentrations increased. The scavenging activity on superoxide radical by CSVC was stronger than that by CS. At low concentrations (<0.05 mg/ml), the scavenging activity of CSVC was stronger than that of VC, but after certain concentrations (>0.1 mg/ml), its scavenging activity was lower than that of VC.

In the present study, water soluble CS-ascorbic acid complex was synthesized by ionic interaction in the heterogeneous condition. Characterizations using FT-IR, DSC and ^1^H-NMR confirmed the formation of complex instead of physical mixture. When compared with CS, the complex showed good water solubility and increased •O2^−^ scavenging capability. Pharmaceutical applications of this complex, needs to be explored further.

## References

[1] Ravi Kumar MN (2000). A review of chitin and CS applications. React Funct Polym.

[2] Agnihotri SA, Mallikarjuna NN, Aminabhavi TM (2004). Recent advances on CS-based micro- and nanoparticles in drug delivery. J Controlled Release.

[3] Dodane V, Vilivalam VD (1998). Pharmaceutical applications of CS. Pharm Sci Technol Today.

[4] Dyer AM, Hinchcliffe M, Watts P (2002). Nasal delivery of insulin using novel CS based formulations: A comparative study in two animal models between simple CS formulations and CS nanoparticles. Pharm Res.

[5] Hoven VP, Tangpasuthadol V, Angkitpaiboon Y, Vallapab N, Kiatkamjornwongc S (2007). Surface-charged CS: Preparation and protein adsorption. Carbohydr Polym.

[6] Wang Y, Nessa BV (1989). Site-specific cleavage of supercoiled DNA by ascorbate/Cu(II). Nucleic Acids Res.

[7] Zhao LH, Qiu JP, Huang M, Yong HJ (2004). Advances in the research of saccharide derivatives of L-Ascorbic acid. Feed Industry.

[8] Dikusar EA, Kozlov NG, Mel'nichuk LA (2004). Salts of L-ascorbic acid with certain substituted amines and triphenylphosphine. Chem Nat Compd.

[9] Yang Y, Liu WS, Han BQ, Sun HZ (2006). Antioxidative Properties of a newly synthesized 2-glucosamine-thiazolidine-4(R)-Carboxylic Acid (GlcNH2Cys) in mice. Nutr Res.

[10] Wu XQ, Qiao W, Yan SJ, Li F, LI NL, Ma L (2007). Synthesis and antioxidant activity evaluation of L-ascorbyl 6-p-hydroxyl benzoate. Acta Scientiarum Naturalium Universitatis Sunyatseni.

[11] Tan ZJ, Zhang H (2002). A comparison of four pyorgallol autoxidation methods on determining superoxide dismutase activity. Acta Scientiarum Naturalium Universitatis Neimongol.

[12] Angerer JD, Cyron DM, Iyer S, Jerrell TA (2001). Dry acid-chitosan complexes. US Patent 6326475.

[13] Capuzzi G, Nostro PL, Kulkarni K, Fernandez JE (1996). Mixtures of stearoyl-6-O-ascorbic acid and α-tocopherol: A monolayer study at the gas/water interface. Langmuir.

[14] Banerjee T, Mitra S, Singh AK, Sharma RK, Maitra A (2002). Preparation, characterization and biodistribution of ultrafine CS nanoparticles. Int J Pharm.

[15] Zheng H, Du YM (2002). Preparation of N-acylCS and structure properties of their films. J Wuhan Univ.

[16] Gao YF, Liu WH, Li ZG, Ma CN (2002). Assignment of infrared spectrum of L-ascorbic acid. Chin J Spectrosc Lab.

[17] Zheng C, Yan X, Si JG, Meng Y, Qi Z, Tao Z (2008). Ionic interactions between sulfuric acid and CS membranes. Carbohydr Polym.

[18] Mao SR, Shuai XT, Unger F, Simona M, Bi DZ, Kissel T (2004). The depolymerization of CS: Effects on physicochemical and biological properties. Int J Pharm.

[19] Yan CY, Chen DW, Gu JW, Hu HY, Zhao XL, Qiao MX (2006). Preparation of N-succinyl-CS and their physical-chemical properties as a novel excipient. Yakugaku Zassi.

[20] Fan L, Wu H, Zhang H, Li F, Yang TH, Gu CH (2008). Novel super pH-sensitive nanoparticles responsive to tumor extracellular pH. Carbohydr Polym.

[21] Champagne LM (2008). The synthesis of water soluble N-acyl CS derivatives for characterization as antibacterial agents.

[22] Zhang YJ, Liu XP (2007). Studies of NMR acquisition parameters for quantitative analysis of drugs. Chin J Magn Reson.

[23] Cravotto G, Daghero P (2008). A method for preparing ascorbic acid or a salt thereof from plant Matrices.

[24] Yui T, Imada K, Okuyama K, Obata Y, Suzuki K, Ogawa K (1994). Molecular and crystal structure of the anhydrous form of CS. Macromolecules.

[25] Tao SN, Xie WM, Xu PX (2004). Superoxide anion scavenging activity of graft CS derivatives. Carbohydr Polym.

